# Non-Surgical Treatment of the Prostate in Emergency Cases1Read at the thirty-eighth annual meeting of the Medical Association of Central New York, held at Buffalo. October 24, 1905.

**Published:** 1906-03

**Authors:** J. Henry Dowd

**Affiliations:** Buffalo, N. Y.


### Non-Surgical Treatment of the Prostate in Emergency Cases.[1]

By J. HENRY DOWD, M. D., Buffalo, N. Y.

IN speaking of disease of the prostate at this time I shall briefly refer to the causes and conditions found in and around this organ in what may be termed emergency cases. I use the term "emergency," wishing only to consider those conditions where prompt and energetic measures must be applied for the relief of retention and pain, as in acute parenchymatous disease and hypertrophy with pain and retention, and in follicular conditions with frequency of desire and pain in micturition. We are all aware that the first and last conditions are due to inflammation and usually complicating gonorrhea, a condition that to some degree, if not a great extent, can be avoided. Hypertrophy is beyond our prevention, but with a little advice, and care on the part of the patient, retention should be avioded.

### ACUTE INFLAMMATORY CONDITIONS.

We must look at our patients suffering with acute inflammatory conditions of the genitourinary tract, the same as we do with acute inflammation elsewhere; that is, no matter how carefully instructions are carried out, in some instances complications will develop. Others, however, though disregarding all advice and no matter how treated, go on to a perfect recovery without the least sign of complication. The predisposing causes of acute prostatitis can be passed over and only the exciting causes considered. Here, as in all other inflamations, infection is the true cause, the infecting agent being derived from either the urethra,

1. Read at the thirty-eighth annual meeting of the Medical Association of Central New York, held at Buffalo, October 24, 1905.

the rectum or the blood; although in the latter, which is generally tubercular, we can scarcely consider the condition acute.

From the urethra we have the gonococci, strepto- and staphylococci and the colon bacilli, and from the rectum the latter only. Although it has been stated that in some individuals complications will not develop no matter what takes place, there is no doubt that indiscretions and unskilful treatment are important factors in those cases that do develop. One thing is a proven fact—namely, that the posterior urethra is involved in every case of gonorrhea, and it is from this portion of the canal that infection spreads to the prostate.

Considering the treatment that this portion of the urethra is submitted to in a vast majority of cases, it is surprising that acute prostatitis does not result in ten times as often as it does. Sexual intercourse and ungratified sexual desire are other very important causes on which too much stress cannot be laid, not only in the production of acute parenchymatous prostatitis, but as predisposing causes to a condition arising later in life and where there may never have been any urethral inflammation. I refer to hypertrophy. In acute conditions although the gland may swell to proportions where complete retention takes place, in the great majority of cases it is due to a swelling of the mucous membrane, either from inflammation or edema or both combined. In many cases I have seen the prostate swollen to the size of a billard ball, yet no retention. On the other hand, retention has taken place where the enlargement was practically nil.

The symptoms of acute parenchymatous prostatitis are very marked. If it arises as a complication of acute gonorrhea, the discharge suddenly stops, pain varying in degree is located in the region of the rectum, and urination varies from slight straining to complete retention. Frequency may not be increased over that which occurs in acute posterior urethritis, but the patient will be disturbed at night from four to six times or even every hour. Examination per rectum reveals a large pulsating swelling just inside the sphincter. In the follicular variety urination is of the same frequency, the discharge lessens in quantity, and there is a perceptible straining but no retention. Examination reveals the prostate only slightly increased in size, though hot and tender.

### HYPERTROPHY.

It is the writer's opinion that in hypertrophy of the prostate we have a certain inflammatory element constantly present, and that the sudden changes that take place are due to increased activity because of that process. Thus, in retention we have practically the same condition present as in acute parenchymatous disease. In the acute condition there is a normal prostate in which infection occurs; whereas, in hypertrophy, inflammation being present, all that is necessary is irritation to cause an exacerbation. Although gonorrhea would be a most important factor, it is rare to meet this disease in prostatics, therefore we must look elsewhere for a cause.

The most important cause of retention, accompanying hypertrophy, is wetting of the feet, chilling of the body and undue nervous excitement from any cause, but especially sexual excess. In contradistinction to the acute condition we here find an elongated and distorted posterior urethra, being due to unsymmetrical enlargement of the lobes and projection into the urethra of individual hypertrophied bodies resembling fibromates.

### TREATMENT.

There is only one method that should be considered in the acute condition, at least in my view, which may be summarised as follows:

1. Stop all local medication.

2. Calomel, gr. ij, followed by a saline in three hours. This can be repeated on the third day.

3. At least six leeches to the perineum, close to the sphincter.

4. After the leeches fall, place the patient in a hot sitz bath for fifteen minutes.

5. Two or three days after the discharge has reappeared, resume local medication, being careful that no solution enters the posterior urethra.

Although the leeches may relieve the retention in the course of a few hours, it may become necessary to use the catheter. Before this instrument is passed the urethra should be carefully irrigated with a mild antiseptic fluid. To a great extent retention of urine is preventable in case of prostatic hypertrophy, by a little advice of the physician and care upon the part of the patient.

Patients with hypertrophied prostates should not expose themselves to sudden changes of the weather, especially to a degree to cause a sudden chilling of the body or wetting of the feet. Great nervous strain should be avoided as much as possible. The use of liquor should be curtailed, and the diet must be void of all articles of food known to cause irritation of the mucous membrane of the urinary tract through its effect on the urine.

The use of the catheter is one of the most important considerations in the care of retention from any cause, but especially in connection with hypertrophy. No instrument should ever be thought of except one having the Mercer curve, and even this should be used with the greatest care. As soon as its forward movement is retarded by any unyielding substance its progress should be stopped.

If these few instructions are carefully carried out, it will be found that one catherisation possibly will be all that is necessary; whereas if traumatism is produced, by steel or improperly curved instruments, the retention may continue for weeks, even ending in abscess, or other serious results.

When the condition is relieved the patient should be given the few general instructions already alluded to, and placed upon some urinary antiseptic in combination with a drug known to have a sedative action upon this region. For this purpose I generally use uriseptin in connection with hyoscyamus. The most satisfactory results are at times derived from these drugs, even one dose in 24 hours.

354 Franklin Street.

